# New Coleoptera records from New Brunswick, Canada: Geotrupidae and Scarabaeidae

**DOI:** 10.3897/zookeys.179.2607

**Published:** 2012-04-04

**Authors:** Reginald P. Webster, Jon D. Sweeney, Ian DeMerchant

**Affiliations:** 1 Natural Resources Canada, Canadian Forest Service - Atlantic Forestry Centre, 1350 Regent St., P.O. Box 4000, Fredericton, NB, Canada E3B 5P7

**Keywords:** Geotrupidae, Scarabaeidae, new records, Canada, New Brunswick

## Abstract

Two species of Geotrupidae, *Geotrupes splendidus splendidus* (Fabricius) and *Odonteus liebecki* (Wallis), are newly reported for New Brunswick, Canada. Twelve species of Scarabaeidae are added to the faunal list of the province, including *Aegialia criddlei* Brown, *Caelius humeralis* (Brown), *Dialytellus dialytoides* (Fall), *Diapterna omissa* (LeConte), *Diapterna pinguis* (Haldeman), *Planolinoides aenictus* (Cooper and Gordon), *Stenotothorax badipes* (Melsheimer), and *Ataenius strigatus* (Say), which are also newly recorded for the Maritime provinces. Collection data, habitat data, and distribution maps are presented for each species.

## Introduction

This paper treats new species records from New Brunswick, Canada in the Coleoptera families Geotrupidae and Scarabaeidae. The Geotrupidae (earth-boring scarab beetles), as their common name implies, are burrowers in soil and they provision the burrows for their larvae with dung, fungi, humus, or dead leaves, depending on the species (Jameson 2002). Adults dig vertical burrows that are 15 to 200 cm in depth, although burrows of some species can extend to 3.0 m in depth. Adults of many species are nocturnal and are often attracted to lights and are saprophagous, coprophagous, mycetophagous, or do not feed as adults (Jameson 2002).

Ratcliffe et al. (2002) provided a general overview of the taxonomy and ecology of the family Scarabaeidae (scarab beetles) of North America, and this reference should be consulted for more details on this family. The Scarabaeidae are very diverse in life histories. Adults, depending on species, feed on dung, carrion, fungi, vegetation, pollen, and a few species live in nests of ants, rodents, or birds (Ratcliffe et al. 2002). Adults in the subfamilies Scarabaeinae and Aphodiinae provision burrows for their larvae; adults in the subfamilies Melolonthinae, Dynastinae, Rutelinae, and Cetoniinae are phytophagous and feed on leaves and fruit (Ratcliffe et al. 2002). Some species occasionally defoliate trees and shrubs. Larvae feed on rotting wood (Dynastinae, Rutelinae) or grass roots (Melolonthinae, Dynastinae, Rutelinae, Cetoniinae). Depending on species, adults are either diurnal or nocturnal, and some nocturnally active species are attracted to lights in large numbers (*Phyllophaga* spp., for example) (Ratcliffe et al. 2002).

Twenty-eight species of Geotrupidae are known from North America (Jameson 2002), and 13 species from Canada (McNamara 1991). Only two species, *Geotrupes balyi* Jekel and the adventive *Geotrupes stercorarius* (Linnaeus) were reported from New Brunswick, Canada by McNamara (1991). Around 1700 species of Scarabaeidae are known from North America (Ratcliffe et al. 2002). McNamara (1991) listed 197 species from Canada, excluding the Ochodaeidae, Glaresidae, Trogidae, Geotrupidae, and Glaphyridae, which are now treated as separate families in the Scarabaeoidea (Ratcliffe et al. 2002). Only 39 species of Scarabaeidae were listed from New Brunswick by McNamara (1991). Here, we newly report two species of Geotrupidae and add 12 species of Scarabaeidae to the faunal list of New Brunswick.

## Methods and conventions

The following records are based on specimens collected during a general survey by the first author to document the Coleoptera fauna of New Brunswick and from by-catch samples obtained during a study to develop a general attractant for the detection of invasive species of Cerambycidae.

### Collection methods

Various methods were employed to collect the species reported in this study. Details are outlined in Webster et al. (2009, Appendix). See Webster et al. (in press) for details of the methods used to deploy Lindgren 12-funnel traps and for sample collection. A description of the habitat was recorded for all specimens collected during this survey. Locality and habitat data are presented exactly as on labels for each record. This information, as well as additional collecting notes, is summarized and discussed in the collection and habitat data section for each species.

### Distribution

Distribution maps, created using ArcMap and ArcGIS, are presented for each species in New Brunswick. Every species is cited with current distribution in Canada and Alaska, using abbreviations for the state, provinces, and territories. New records for New Brunswick are indicated in bold under Distribution in Canada and Alaska. The following abbreviations are used in the text:

**Table:** 

**AK**	Alaska	**MB**	Manitoba
**YT**	Yukon Territory	**ON**	Ontario
**NT**	Northwest Territories	**QC**	Quebec
**NU**	Nunavut	**NB**	New Brunswick
**BC**	British Columbia	**PE**	Prince Edward Island
**AB**	Alberta	**NS**	Nova Scotia
**SK**	Saskatchewan	**NF & LB**	Newfoundland and Labrador

*Newfoundland and Labrador are each treated separately under the current Distribution in Canada and Alaska.

Acronyms of collections examined or where specimens reside referred to in this study are as follows:

AFCAtlantic Forestry Centre, Natural Resources Canada, Canadian Forest Service, Fredericton, New Brunswick, Canada


CNCCanadian National Collection of Insects, Arachnids and Nematodes, Agriculture and Agri-Food Canada, Ottawa, Ontario, Canada


NBMNew Brunswick Museum, Saint John, New Brunswick, Canada


RWCReginald P. Webster Collection, Charters Settlement, New Brunswick, Canada


## Results

Here, we newly report two species of Geotrupidae and 12 species of Scarabaeidae for New Brunswick, Canada. *Aegialia criddlei* Brown, *Ataenius strigatus* (Say), *Caelius humeralis* (Brown), *Dialytellus dialytoides* (Fall), *Diapterna omissa* (LeConte), *Diapterna pinguis* (Haldeman), *Planolinoides aenictus* (Cooper and Gordon), and *Stenotothorax badipes* (Melsheimer) are newly recorded for the Maritime provinces (Table 1).

**Table 1.** Species of Geotrupidae and Scarabaeidae reported from New Brunswick, Canada.

Family Geotrupidae Latreille
Subfamily Bolboceratinae Mulsant
Tribe Bolboceratini Mulsant
*Odonteus liebecki* (Wallis)*
Subfamily Geotrupinae Latreille
Tribe Geotrupini Latreille
*Geotrupes balyi* Jekel
*Geotrupes splendidus splendidus* (Fabricius)*
*Geotrupes stercorarius* (Linnaeus)
Family Scarabaeidae Latreille
Subfamily Aegialiinae Laporte
*Aegialia blanchardi* Horn
*Aegialia criddlei* Brown**
*Aegialia lacustris* LeConte
*Aegialia nana* Brown
*Aegialia opifex* Horn*
*Caelius humeralis* (Brown)
*Caelius rufescens* (Horn)
Subfamily Aphodiinae Leach
Tribe Aphodiini Leach
*Acrossus rubripennis* (Horn)
*Agoliinus guttatus* (Eschscholtz)
*Agoliinus leopardus* (Horn)
*Agoliinus manitobensis* (Brown)
*Aphodius fimetarius* (Linnaeus)
*Calamosternus granarius* (Linnaeus)
*Chilothorax distinctus* (Müller)
*Colobopterus erraticus* (Linnaeus)
*Dialytellus dialytoides* (Fall)*
*Dialytes striatulus* (Say)
*Diapterna hyperborea* (LeConte)
*Diapterna omissa* (LeConte)*
*Diapterna pinguis* (Haldeman)**
*Eupleurus subterraneus* (Linnaeus)
*Melinopterus prodromus* (Brahm)
*Oscarinus rusicola* (Melsheimer)
*Otophorus haemorrhoidalis* (Linnaeus)
*Planolinellus vittatus* (Say)
*Planolinoides aenictus* (Cooper & Gordon)*
*Planolinoides borealis* (Gyllenhal)
*Planolinus tenellus* (Say)
*Stenotothorax badipes* (Melsheimer)*
*Teuchestes fossor* (Linnaeus)
*Trichonotulus scrofa* (Fabricius)
Tribe Euparini Schmidt
*Ataenius abditus* (Haldeman)
*Ataenius strigatus* (Say)*
Subfamily Scarabaeinae Latreille
Tribe Onthophagini Burmeister
*Onthophagus hecate* (Panzer)
*Onthophagus nuchicornis* (Linnaeus)
Subfamily Melolonthinae Leach
Tribe Diplotaxini Kirby
*Diplotaxis tristis* Kirby
Tribe Hopliini Latreille
*Hoplia trifasciata* Say
Tribe Dichelonychini Burmeister
*Dichelonyx albicollis* Burmeister
*Dichelonyx diluta* (Fall)
*Dichelonyx elongatula* (Schonherr)
*Dichelonyx subvittata* LeConte
Tribe Melolonthini Leach
*Phyllophaga anxia* (LeConte)
*Phyllophaga drakii* (Kirby)
*Phyllophaga futilis* (LeConte)
Tribe Sericini Kirby
*Serica atracapilla* (Kirby)
*Serica georgiana* Leng
*Serica tristis* LeConte
Subfamily Dynastinae MacLeay
Tribe Pentodontini Mulsant
*Tomarus relictus* (Say)
Subfamily Cetoniinae Leach
Tribe Cremastocheilini Burmeister & Schaum
*Cremastocheilus castaneus* Knoch*
Tribe Trichiini Fleming
*Gnorimella maculosa* (Knoch)*
*Osmoderma scabra* (Palisot de Beauvois)
*Osmoderma eremicola* (Knoch)*
*Trichiotinus assimilis* (Kirby)

Notes: *New to province, **New to Maritime provinces.

### Species accounts

All records below are species newly recorded for New Brunswick, Canada. Species followed by ** are newly recorded from the Maritime provinces (New Brunswick, Nova Scotia, Prince Edward Island) of Canada.

The classification of the Geotrupidae and Scarabaeidae follows Bouchard et al. (2011).

#### Family Geotrupidae Latreille, 1802

Subfamily Bolboceratinae Mulsant, 1842

Tribe Bolboceratini Mulsant, 1842

##### Odonteus liebecki

(Wallis, 1928)

http://species-id.net/wiki/Odonteus_liebecki

Map 1

###### Material examined.

**New Brunswick, York Co.**, Charters Settlement, 45.8395°N, 66.7391°W, 10.VI.2007, 25.VI.2009, R. P. Webster, mixed forest, u.v. light (2, RWC).

###### Collection and habitat data.

Both individuals of this species were collected during June at an ultraviolet light deployed near a mixed forest.

###### Distribution in Canada and Alaska.

ON, QC, **NB**, NS (McNamara 1991).

**Map 1.**
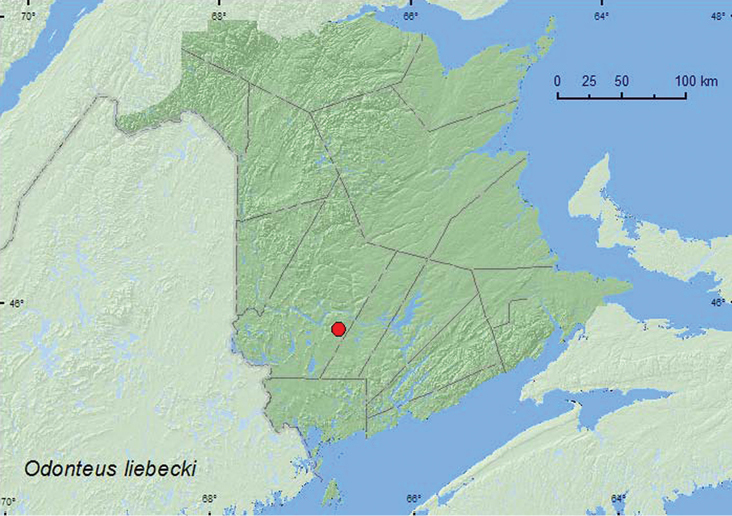
Collection localities in New Brunswick, Canada of *Odonteus liebecki*.

##### Subfamily Geotrupinae Latreille, 1802

Tribe Geotrupini Latreille, 1802

###### Geotrupes splendidus splendidus

(Fabricius, 1775)

http://species-id.net/wiki/Geotrupes_splendidus_splendidus

Map 2

####### Material examined.

**New Brunswick, Queens Co.**, Cranberry Lake P.N.A. (Protected Natural Area), 46.1125°N, 65.6075°W, 11-18.VI.2009, R. Webster & M.-A. Giguère, old red oak forest, Lindgren funnel trap (1, RWC). **York Co.**, Charters Settlement, 45.8428°N, 66.7279°W, 16.IX.2004, 23.IX.2009, R. P. Webster, regenerating mixed forest, baited with pile of decaying mushrooms (2, RWC); Charters Settlement, 45.8395°N, 66.7391°W, 9.IX.2007, R. P. Webster, mixed forest, in decaying (mouldy) corncobs and cornhusks (1, RWC); Canterbury, near Browns Mountain Fen, 45.8964°N, 67.6273°W, 8.IX.2007, R. P. Webster, in flight along woodland trail (1, RWC).

####### Collection and habitat data.

Adultshave been reported from fungi, dung, and various decaying organic material (Howden 1955; Downie and Arnett 1996). Adults from New Brunswick were collected from decaying mushrooms and decaying moldy corncobs and cornhusks. One individual was captured in a Lindgren funnel trap deployed in an old red oak (*Quercus rubra* L.) forest, and another was collected as it flew along a woodland trail. Adults were collected during June and September.

####### Distribution in Canada and Alaska.

ON, QC, **NB**, NS (McNamara 1991).

**Map 2.**
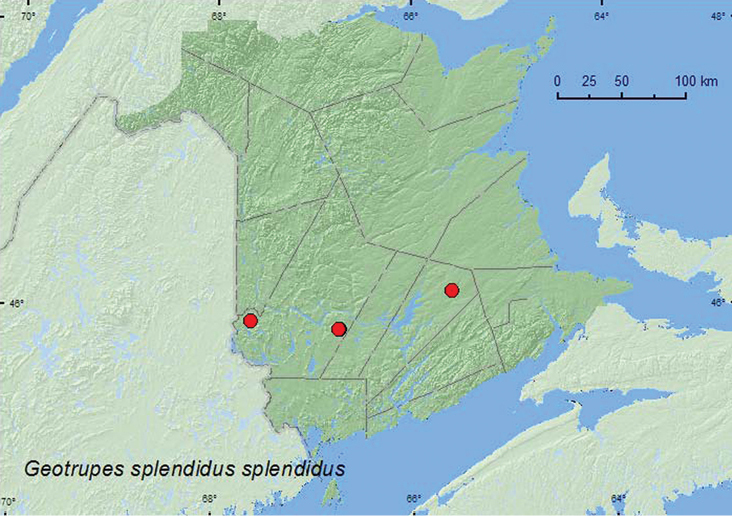
Collection localities in New Brunswick, Canada of *Geotrupes splendidus splendidus*.

#### Family Scarabaeidae Latreille, 1802

Subfamily Aegialiinae Laporte, 1840

##### Aegialia criddlei

Brown, 1931**

http://species-id.net/wiki/Aegialia_criddlei

Map 3

###### Material examined.

**New Brunswick, Albert Co.**, Waterside, Waterside Beach, 45.6282°N, 64.8129°W, 29.V.2010, R. P. Webster & M.-A. Giguère, sea beach, white sand, under log (6, RWC).

###### Collection and habitat data.

No habitat data were reported by Brown (1931) or Gordon and Cartwright (1988) for this species. The *Aegialia* (sensu stricto) are usually found on coastal and inland dune systems or on gravel shores of streams and ponds (subgenus *Psammoporus*) (Gordon and Cartwright 1988). The adults from New Brunswick were found under driftwood on a sand dune along a sea beach. Adults were collected during late May.

###### Distribution in Canada and Alaska.

AK, BC, AB, SK, MB, ON, QC, **NB**, NF (McNamara 1991).

**Map 3.**
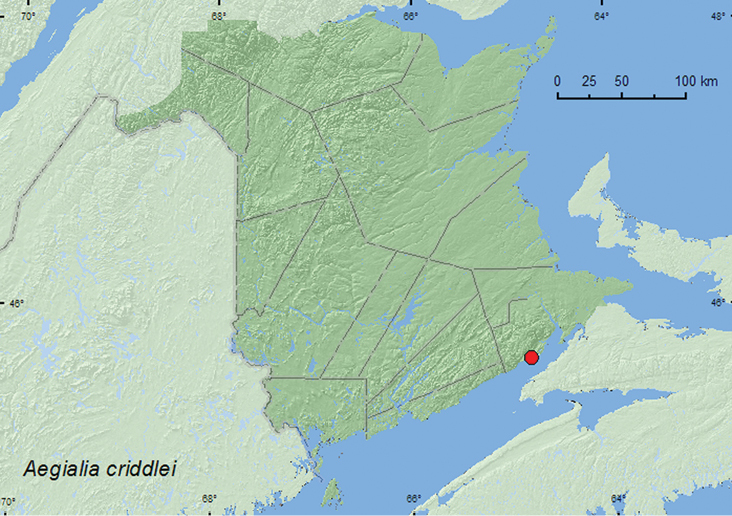
Collection localities in New Brunswick, Canada of *Aegialia criddlei*.

##### Aegialia opifex

Horn, 1887

http://species-id.net/wiki/Aegialia_opifex

Map 4

###### Material examined.

**New Brunswick, Queens Co.**, Bayard, at Nerepis River, 45.4473°N, 66.3318°W, 24.V.2009, R. P. Webster, river margin on sand bar, under log set in sand (1, RWC).

**Map 4.**
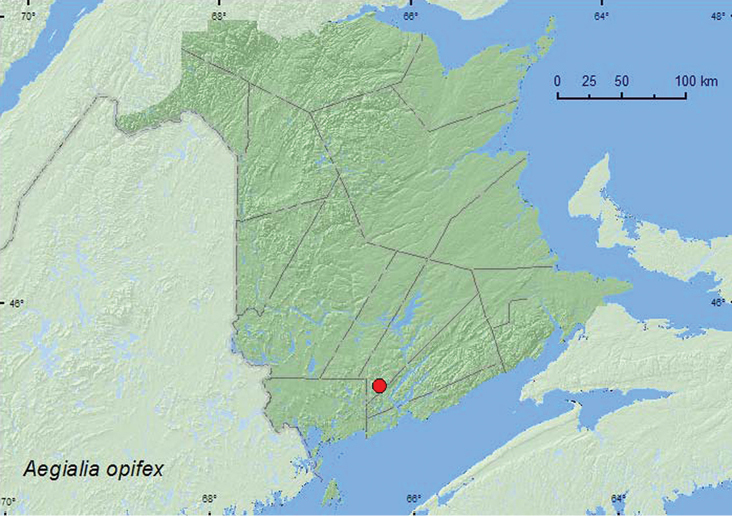
Collection localities in New Brunswick, Canada of *Aegialia opifex*.

###### Collection and habitat data.

No habitat data on this species were included in Gordon and Cartwright (1988). The specimen from New Brunswick was found under a log set in sand on a sand bar during late May.

###### Distribution in Canada and Alaska.

ON, QC, **NB**, NS, PE (McNamara 1991).

##### Caelius humeralis

(Brown, 1931)**

http://species-id.net/wiki/Caelius_humeralis

Map 5

###### Material examined.

**New Brunswick, Carleton Co.**, Jackson Falls, Bell Forest, 46.2200°N, 67.7231°W, 12–19.VI.2008, 19–27.VI.2008, R. P. Webster, mature hardwood forest, Lindgren funnel traps (2, RWC); same locality but 46.2150°N, 67.7190°W, 2.VI.2005, M.-A. Giguère & R. Webster, floodplain forest with butternut, adult collected while in flight (1, RWC). **Restigouche Co.**, Dionne Brook P.N.A., 47.9064°N, 68.3441°W, 31.V–15.VI.2011, M. Roy & V. Webster, old-growth white spruce and balsam fir forest, Lindgren funnel trap (1, RWC).

###### Collection and habitat data.

No habitat information was included for this species in Gordon and Cartwright (1988). In New Brunswick, adults were captured in Lindgren funnel traps deployed in hardwood forest and an old-growth white spruce and balsam fir forest. One individual was collected with an aerial net during an evening flight near a floodplain forest. Adults were collected during May and June.

###### Distribution in Canada and Alaska.

ON, QC, **NB** (McNamara 1991).

**Map 5.**
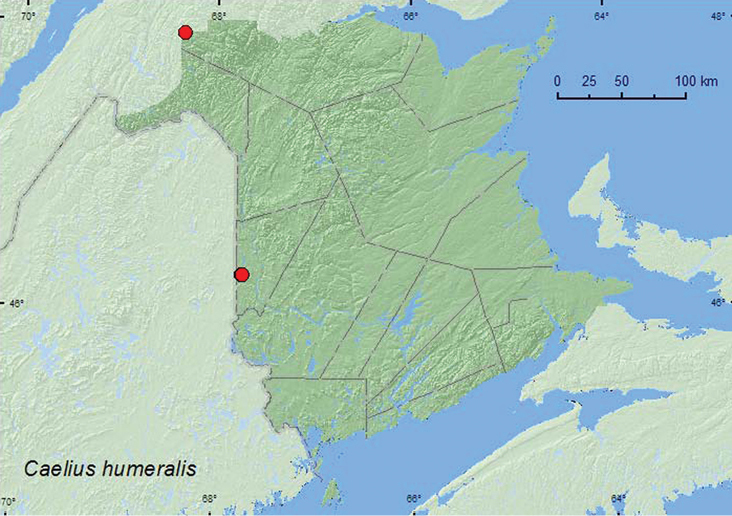
Collection localities in New Brunswick, Canada of *Caelius humeralis*.

##### Subfamily Aphodiinae Leach, 1815

Tribe Aphodiini Leach, 1815

###### Dialytellus dialytoides

(Fall, 1907)**

http://species-id.net/wiki/Dialytellus_dialytoides

Map 6

####### Material examined.

**New Brunswick, Carleton Co.**, Meduxnekeag Valley Nature Preserve, 46.1940°N, 67.6801°W, 12.VIII.2004, 31.VIII.2006, R. P. Webster, hardwood forest, in decaying mushrooms (2, RWC). **York Co.**,Charters Settlement, 45.8428°N, 66.7275°W, 6.X.2005, R. P. Webster, regenerating mixed forest, baited with pile of decaying mushrooms (1, RWC); same locality and collector but 45.8286°N, 66.7365°W, 15.VIII.2004, regenerating mixed forest, baited with pile of decaying mushrooms (3, RWC).

####### Collection and habitat data.

*Dialytellus dialytoides* is usually associated with deer (*Odocoileus virginianus* (Zimmerman)) dung in forests or in damp soil under deer dung, although two large series were taken from rotting mushrooms in Quebec and Ontario (Gordon and Skelley 2007). Gordon and Skelley (2007) considered the latter records as surprising, but suggested that this might be a survival tactic when the preferred food was not available. In New Brunswick, all specimens were taken from decaying mushrooms. Adults were taken during August and October.

####### Distribution in Canada and Alaska.

ON, QC, **NB** (McNamara 1991).

**Map 6.**
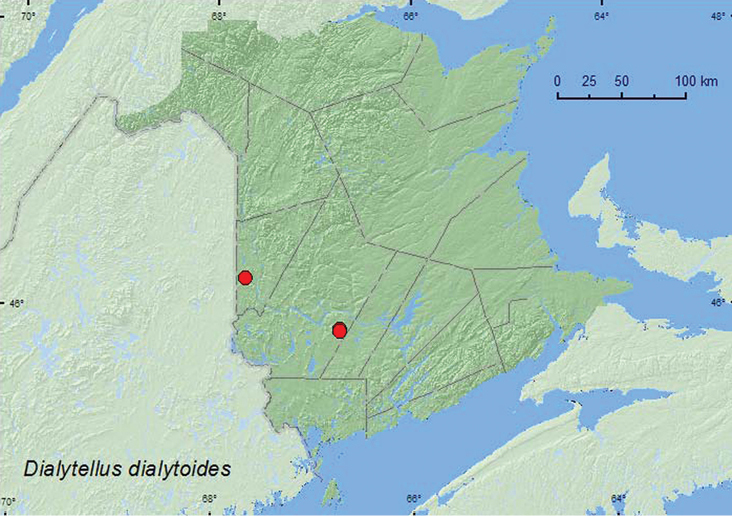
Collection localities in New Brunswick, Canada of *Dialytellus dialytoides*.

###### Diapterna omissa

(LeConte, 1850)**

http://species-id.net/wiki/Diapterna_omissa

Map 7

####### Material examined.

**New Brunswick, York Co.**, Slagundy Dry Ponds, 45.8596°N, 67.1849°W, 8.VII.2006, R. P. Webster, large vernal pond, in moist leaves on pond margin (1, RWC).

####### Collection and habitat data.

Gordon and Skelley (2007) noted that this species was restricted to pond and swamp margins and was likely a detritivore. The sole specimen from New Brunswick was sifted from moist leaves on the margin of a large vernal pond during July.

####### Distribution in Canada and Alaska.

YK, NT, BC, AB, SK, MB, ON, **NB** (McNamara 1991).

**Map 7.**
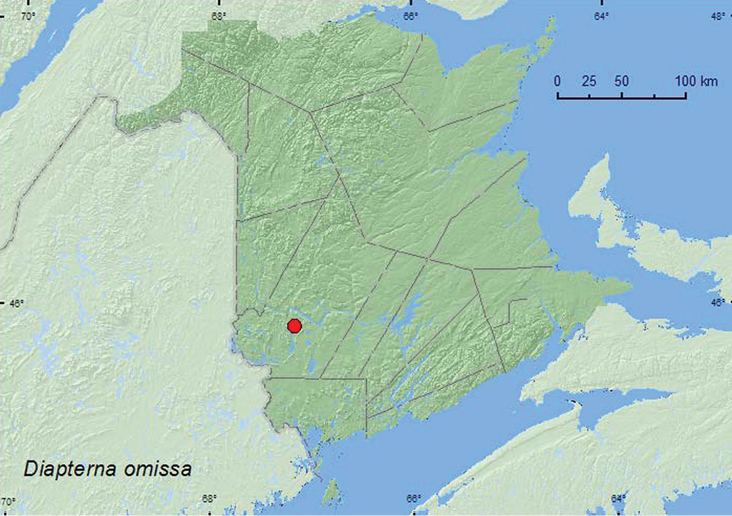
Collection localities in New Brunswick, Canada of *Diapterna omissa*.

###### Diapterna pinguis

(Haldeman, 1848)**

http://species-id.net/wiki/Diapterna_pinguis

Map 8

####### Material examined.

**New Brunswick, Queens Co.**, Cranberry Lake P.N.A, 46.1125°N, 65.6075°W, 29.VI-7.VII.2011, M. Roy & V. Webster, old red oak forest, Lindgren funnel trap (1, RWC).

####### Collection and habitat data.

*Diapterna pinguis* is apparently a detritivore, having been collected in pitfall traps in areas without mammal dung (Gordon and Skelley 2007). It is common “in shelter belts in floodplain forests, apparently feeding in the humus layer” (Helgesen and Post 1967). The individual from New Brunswick was captured during July in a Lindgren funnel trap deployed in an old red oak forest.

####### Distribution in Canada and Alaska.

NT, AB, SK, MB, ON, QC, **NB,** NF (McNamara 1991).

**Map 8.**
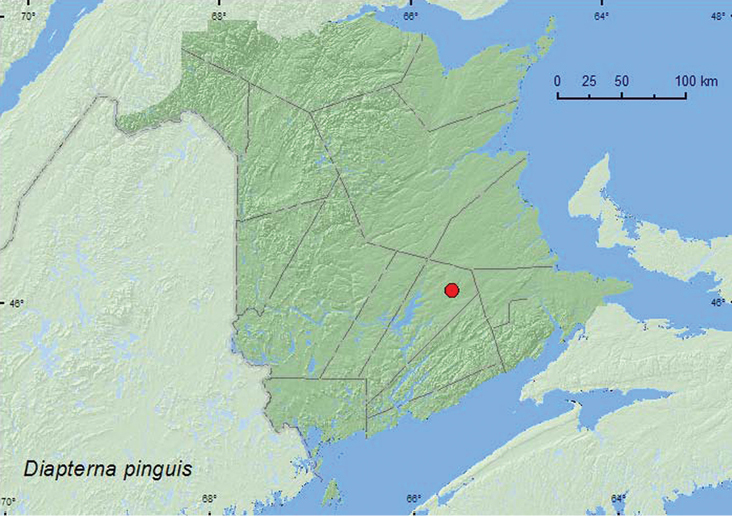
Collection localities in New Brunswick, Canada of *Diapterna pingius*.

###### Planolinoides aenictus

(Cooper and Gordon, 1987)**

http://species-id.net/wiki/Planolinoides_aenictus

Map 9

####### Material examined.

**New Brunswick, Restigouche Co.**, Little Tobique River near Red Brook, 47.4462°N, 67.0689°W, 24.V.2007, R. P. Webster, old growth eastern white cedar swamp, in moss and leaf litter near brook (1, RWC). **Saint John Co.**, Chance Harbour off Rt. 790, 45.1355°N, 66.3673°W, 15.V.2006, R. P. Webster, eastern white cedar swamp, in moss and leaf litter near brook (1, RWC). **York Co.**, New Maryland, Charters Settlement, 45.8430°N, 66.7275°W, 5.V.2006, R. P. Webster, mixed forest, entrance to porcupine den, in porcupine dung (1, RWC).

####### Collection and habitat data.

This species was reported from moose (*Alces alces* (L.)) dung and carnivore scats in a spruce and sphagnum bog in Ontario as well as from localities in Quebec (Cooper and Gordon 1987). Specimens from New Brunswick were sifted from moss and leaf litter in old-growth eastern white cedar (*Thuja occidentalis* L.) swamps and from porcupine (*Erethizon dorsatum* (L.)) dung in the entrance of a porcupine den. All adults were collected during May.

####### Distribution in Canada and Alaska.

ON, **NB**, QC (McNamara 1991; Cooper and Gordon 1987).

**Map 9.**
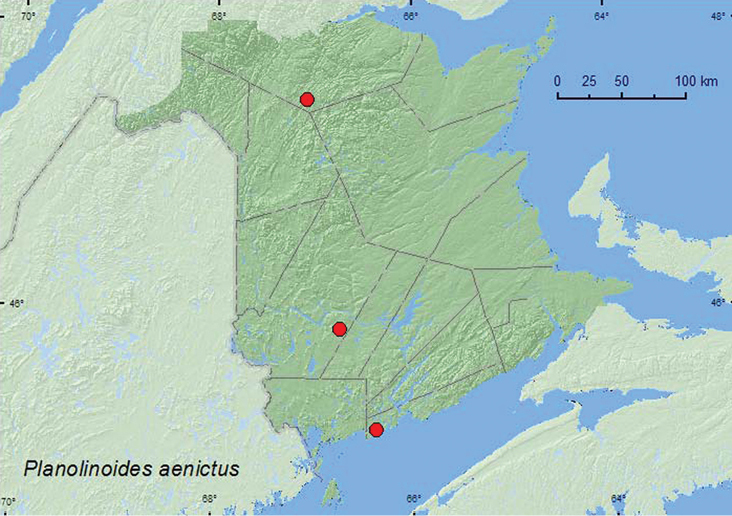
Collection localities in New Brunswick, Canada of *Planolinoides aenictus*.

###### Stenotothorax badipes

(Melsheimer, 1845)**

http://species-id.net/wiki/Stenotothorax_badipes

Map 10

####### Material examined.

**New Brunswick, Queens Co.**, ca. 3.5 km W of Lower Gagetown, 45.7500°N, 66.1833°W, 17.VI.2009, S. Makepeace & R. Webster, in nest contents of barred owl, relatively dry humus-like soil with oak leaves, no urine smell (4, RWC); Cranberry Lake P.N.A, 46.1125°N, 65.6075°W, 24.IV–5.V.2009, R. Webster & M.-A. Giguère, old red oak forest, Lindgren funnel trap (1, AFC). **York Co.**, Keswick Ridge, 46.0040°N, 66.8776°W, 23.V.2006, S. Makepeace, barred owl nest box with 440 gm chicks, in moist nest material with insect parts and small bones (urine smell) (1, RWC).

####### Collection and habitat data.

*Stenotothorax badipes* is usually found in nests of such squirrels as the southern flying squirrel (*Glaucomys volans* (Linnaeus)), the gray squirrel (*Sciurus carolinensis* Gremlin), and the fox squirrel (*Sciurus niger* (Linnaeus)), nesting in tree holes filled with pieces of acorns, detritus, and likely squirrel scat (Gordon and Skelley 2007). The adults from New Brunswick were collected from the contents of barred owl (*Strix varia* Barton) nests that were in either artificial nest boxes or in natural tree cavities (tree holes). The nest material from one nest consisted of relatively dry humus-like soil with oak leaves; the nest material from the other nest was moist and had insect parts and small bones. One specimen was captured in a Lindgren funnel deployed in an old red oak forest. Adults were collected during April, May, and June in New Brunswick.

####### Distribution in Canada and Alaska.

ON, QC, **NB** (McNamara 1991).

**Map 10.**
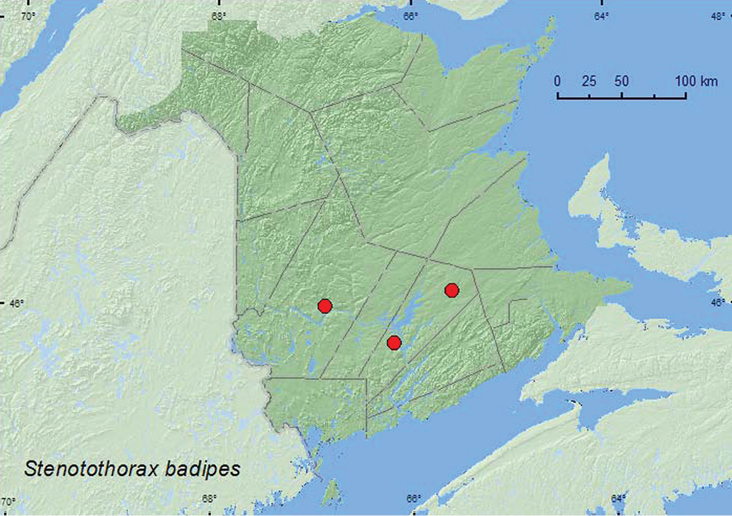
Collection localities in New Brunswick, Canada of *Stenotothorax badipes*.

##### Tribe Eupariini Schmidt, 1910

###### Ataenius strigatus

(Say, 1823)**

http://species-id.net/wiki/Ataenius_strigatus

Map 11

####### Material examined.

**New Brunswick, York Co.**, Charters Settlement, 45.8395°N, 66.7391°W, 10.VI.2007, R. P. Webster, mixed forest, u.v. light (1, RWC).

####### Collection and habitat data.

Nothing has been published on the habitat requirements of this species. The only specimen from New Brunswick was collected at an ultraviolet light during June near a mixed forest.

####### Distribution in Canada and Alaska.

ON, QC, **NB** (McNamara 1991).

**Map 11.**
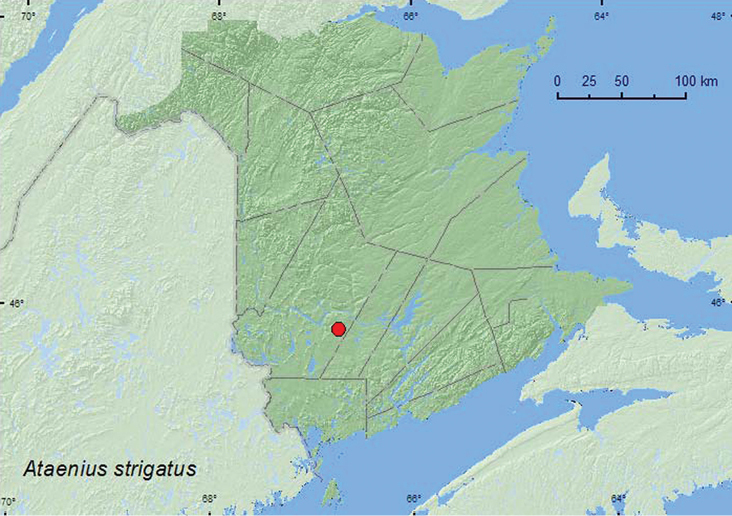
Collection localities in New Brunswick, Canada of *Ataenius strigatus*.

##### Subfamily Cetoniinae Leach, 1815

Tribe Cremastocheilini Burmeister and Schaum, 1841

###### Cremastocheilus castaneus

Knoch, 1801

http://species-id.net/wiki/Cremastocheilus_castaneus

Map 12

####### Material examined.

**New Brunswick, Gloucester Co.**, Bathurst, Daly Point Reserve, 16.V.1994, 28.VII.1998, R. P. Webster, old field, pitfall traps (2, RWC).

####### Collection and habitat data.

Two individuals were collected in pitfall traps in an old field with sandy soil. Adults were collected during May and July.

####### Distribution in Canada and Alaska.

AB, SK, MB, **NB**, NS (McNamara 1991).

**Map 12.**
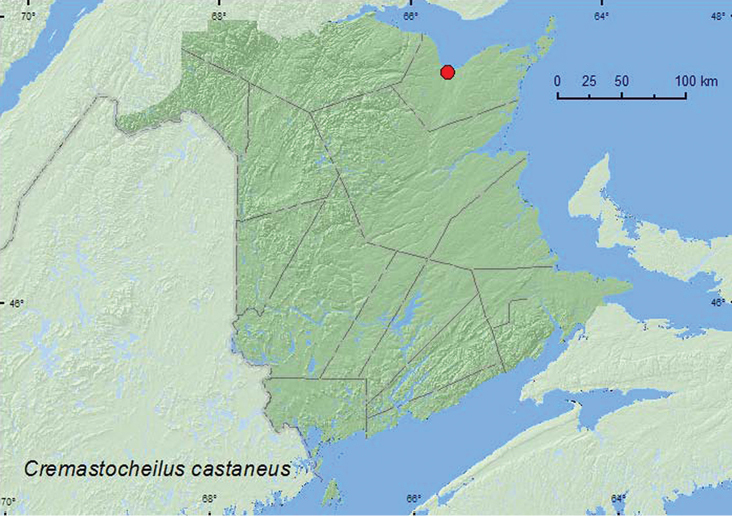
Collection localities in New Brunswick, Canada of *Cremastocheilus castaneus*.

##### Tribe Trichiini Fleming, 1821

###### Gnorimella maculosa

(Knoch, 1801)

http://species-id.net/wiki/Gnorimella_maculosa

Map 13

####### Material examined.

**New Brunswick, Queens Co.**, Grand Lake Meadows P.N.A., 45.8227°N, 66.1209°W, 31.V-15.VI.2010, 15–29.VI.2010, R. Webster & C. MacKay., old silver maple forest with green ash and seasonally flooded marsh, Lindgren funnel traps (10, AFC, RWC); same locality data and forest type, 3–21.VI.2011, 21.VI–5.VII.2011, 5–19.VII.2011, R. Roy & V. Webster, Lindgren funnel traps (20, AFC, NBM, RWC).

####### Collection and habitat data.

Adults of *Gnorimella maculosa* are often found nectaring on flowers and frequent forested areas (See Majka 2010 for a list of plant species and associated references on which adults have been found). In New Brunswick, 30 individuals of *Gnorimella maculosa* were captured in Lindgren funnel traps deployed in an old silver maple (*Acer saccharinum* L.) swamp during June and July in 2010 and 2011.

####### Distribution in Canada and Alaska.

ON, QC, **NB**, NS (McNamara 1991; Majka 2010). Majka (2010) recorded this species for the first time from Nova Scotia and the Maritime provinces based on a specimen from Annapolis Co., Annapolis Royal, collected by Sheilagh Hunt and Christopher G. Majka.

**Map 13.**
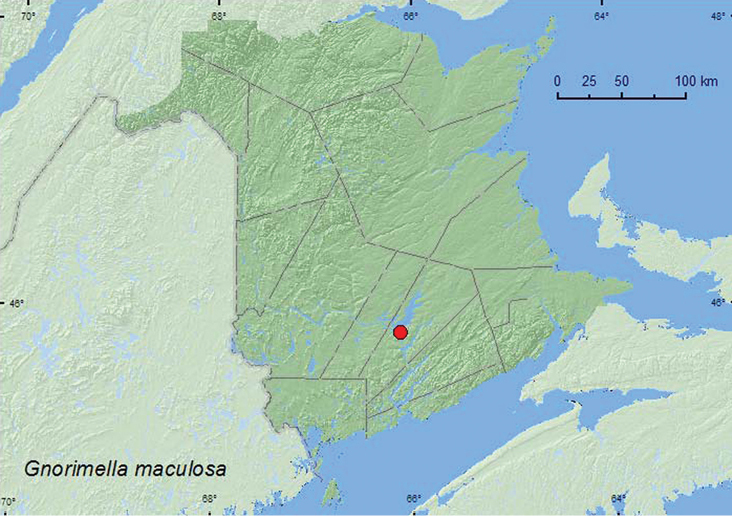
Collection localities in New Brunswick, Canada of *Gnorimella maculosa*.

###### Osmoderma eremicola

(Knoch, 1801)

http://species-id.net/wiki/Osmoderma_eremicola

Map 14

####### Material examined.

**New Brunswick, Queens Co.**, Central Hampstead, 45.6575°N, 66.1412°W, 13.VII.2006, S. Makepeace & R. Webster, hardwood ridge, in nest of barred owl in tree hole (1, RWC); Central Hampstead, 13.VIII.2007, S. Makepeace, near house (1,RWC); Grand Lake Meadows P.N.A., 45.8227°N, 66.1209°W, 29.VI–12.VII.2010, R. Webster, C. MacKay, M. Laity, & R. Johns, old silver maple forest with green ash and seasonally flooded marsh, Lindgren funnel trap (1, AFC); same locality data and forest type, 19.VII–5.VIII.2011, 5-17.VIII.2011, 17–30.VIII.2011, M. Roy & V. Webster, Lindgren funnel traps in forest canopy (14, AFC, NBM, RWC). **York Co.**, Skiff Lake, 15.VIII.1962 (1, AFC); Fredericton, Smythe St. extension, 5.VIII.1945, F. G. Cuming (1 AFC); Fredericton, 8.VIII.1973, 2.VIII.1977 (2, AFC); Douglas, 24.VII.1975 (1, AFC); Charters Settlement, 45.8395°N, 66.7391°W, 30.VII.1993, 13.VIII.2004, R. P. Webster (on ground near house) (2, NBM, RWC).

####### Collection and habitat data.

Larvae of *Osmoderma* species live in decaying wood in the heart of trunks and branches of old and often declining hardwood trees (Packard 1890; Hoffman 1939). *Osmoderma eremicola* with habitat data were collected from the nest contents of a barred owl nesting in a tree hole and on the ground near homesteads. Most (14) individuals were captured in Lindgren funnel traps deployed in the mid canopy of large silver maples in an old silver swamp. Adults were collected during July and August.

####### Distribution in Canada and Alaska.

ON, QC, **NB**, NS (McNamara 1991).

**Map 14.**
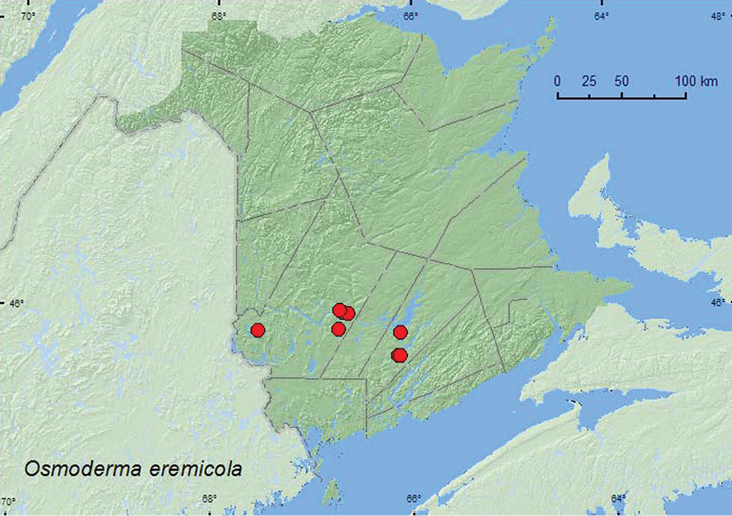
Collection localities in New Brunswick, Canada of *Osmoderma eremicola*.

## Supplementary Material

XML Treatment for Odonteus liebecki

XML Treatment for Geotrupes splendidus splendidus

XML Treatment for Aegialia criddlei

XML Treatment for Aegialia opifex

XML Treatment for Caelius humeralis

XML Treatment for Dialytellus dialytoides

XML Treatment for Diapterna omissa

XML Treatment for Diapterna pinguis

XML Treatment for Planolinoides aenictus

XML Treatment for Stenotothorax badipes

XML Treatment for Ataenius strigatus

XML Treatment for Cremastocheilus castaneus

XML Treatment for Gnorimella maculosa

XML Treatment for Osmoderma eremicola
